# Unmasking the Stenosis: Simultaneous Mitral Valve Replacement and Scimitar Vein Rerouting in a Dextro-Rotated Heart

**DOI:** 10.7759/cureus.106248

**Published:** 2026-03-31

**Authors:** Vimlendra K Chaudhary, Deepak G, Sowmya Ramanan, Baiju Sasi Dharan

**Affiliations:** 1 Department of Cardiovascular and Thoracic surgery, Sree Chitra Tirunal Institute for Medical Sciences and Technology, Thiruvananthapuram, IND

**Keywords:** case report, mitral valve replacement, mitral valve stenosis, rheumatic heart disease, scimitar syndrome

## Abstract

Scimitar syndrome (SS) is a rare congenital condition marked by partial anomalous pulmonary venous connection, dextroposition of the heart, right lung hypoplasia, and systemic arterial supply to the right lung. Also known as congenital veno-lobar syndrome and other names, it has been classified into three types by Lupius et al. A significant number of cases are associated with congenital heart anomalies, while association with acquired cardiac disease is rare. We present the rare case of a 39-year-old woman with exertional dyspnoea during pregnancy, initially diagnosed with mild mitral stenosis, later found to have SS with rheumatic heart disease.

## Introduction

The incidence of scimitar syndrome (SS) is approximately one to three per 100,000 live births [1]. A significant proportion of individuals with Scimitar syndrome, ranging from 19% to 31%, present with associated cardiac anomalies [2]. Association with acquired cardiac disease is even rarer [3]. It involves a partial anomalous pulmonary venous connection, typically where right pulmonary veins drain into the inferior vena cava (IVC), the IVC-right atrial junction, or the right atrium (RA). The scimitar vein drains the entire right lung in two-thirds of the cases, only the lower lobe in one-third, and rarely the left lung [4].

Scimitar syndrome presents bimodally: pulmonary artery hypertension, heart failure, and high mortality, or a milder childhood/adult form often detected incidentally. Or, a severe infantile form with anomalies such as abnormal right lung lobation and right lung hypoplasia (100% incidence rate), dextroposition of the heart (60% incidence rate), hypoplasia of the right pulmonary artery (60% incidence rate), systemic arterial blood supply to the right lower lung from the infra-diaphragmatic aorta (60% incidence rate), ostium secundum atrial septal defect (40% (80% to 90% in infantile form) incidence rate), and right-sided diaphragmatic hernia (15% incidence rate) [5]. On chest X-ray, the anomalous venous shadow resembles a curved 'scimitar' or Turkish sword, giving the syndrome its name [6]. Echocardiography, CT, and MRI play a vital role in delineating the anatomy. Cardiac catheterization further confirms the diagnosis and identifies any anomalous systemic arterial supply to the lung [6]. Indian literature reveals only one case of coexistence of SS with rheumatic heart disease (RHD) [3].

## Case presentation

A 39-year-old woman experienced dyspnea on exertion (DOE) during pregnancy two years prior to presentation. Transthoracic echocardiography revealed features of RHD with mild mitral stenosis. She was started on oral penicillin prophylaxis but was lost to follow-up. She presented to our center as her DOE worsened over the past year.

**Figure 1:**
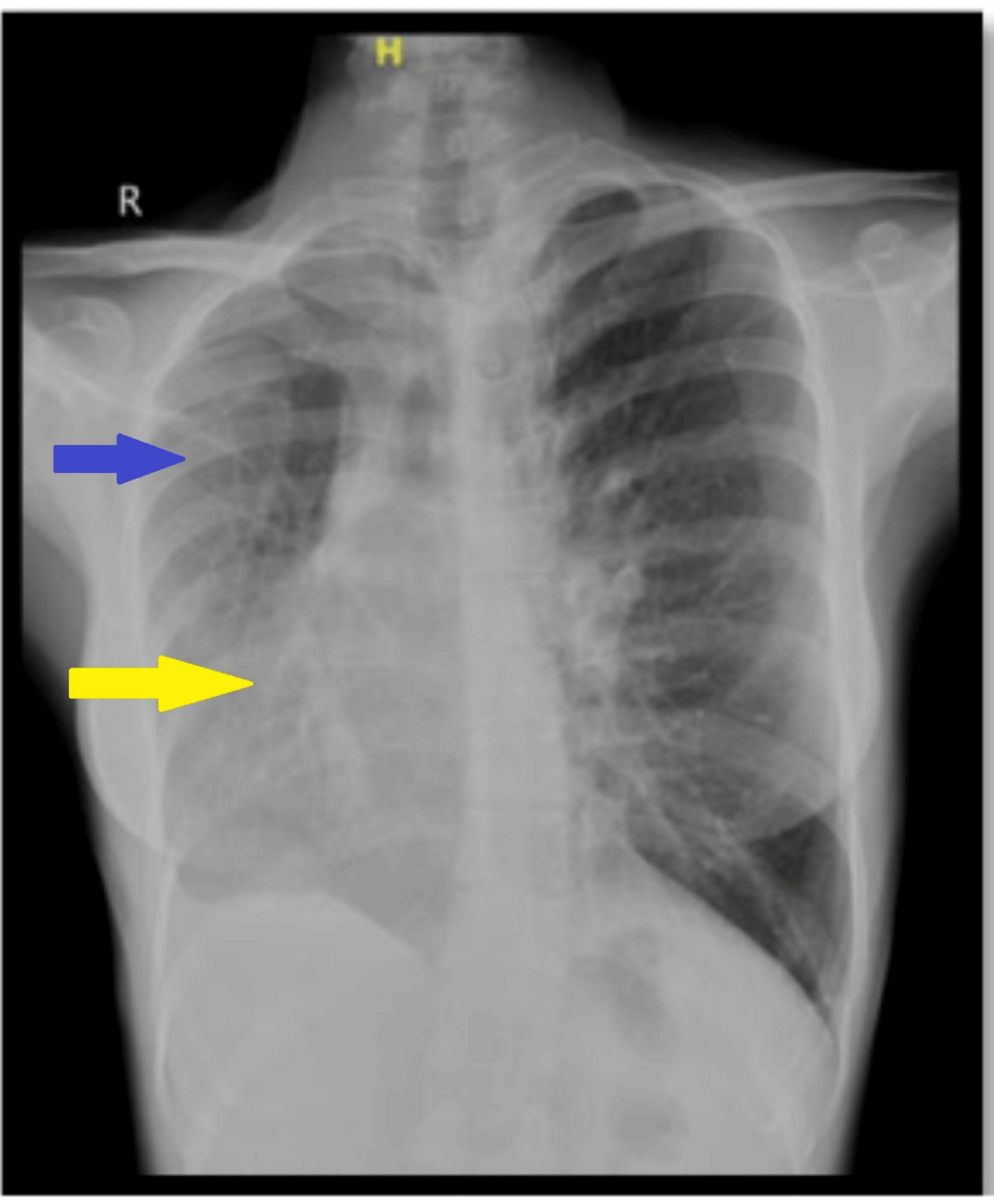
Chest X-ray- showing right-rotated heart (yellow arrow) with hypoplastic right lung field (blue arrow)

**Figure 2:**
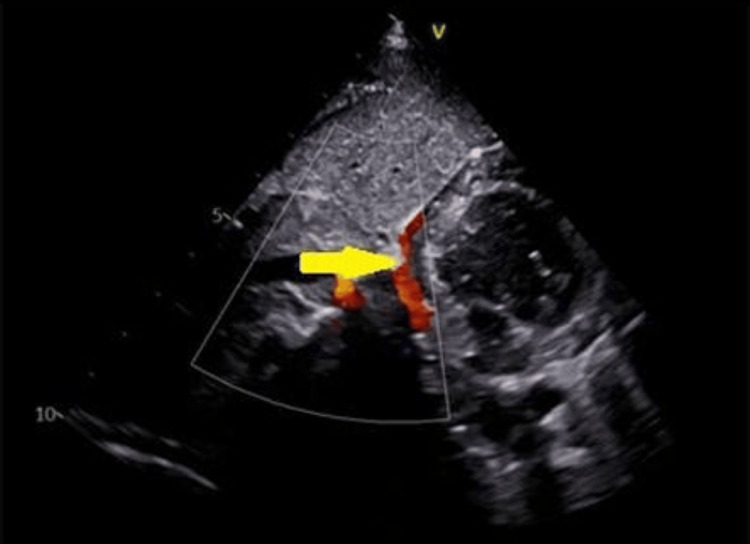
Scimitar vein opening at the IVC-RA junction (yellow arrow) IVC: Inferior vena cava, RA: Right atrium

**Figure 3:**
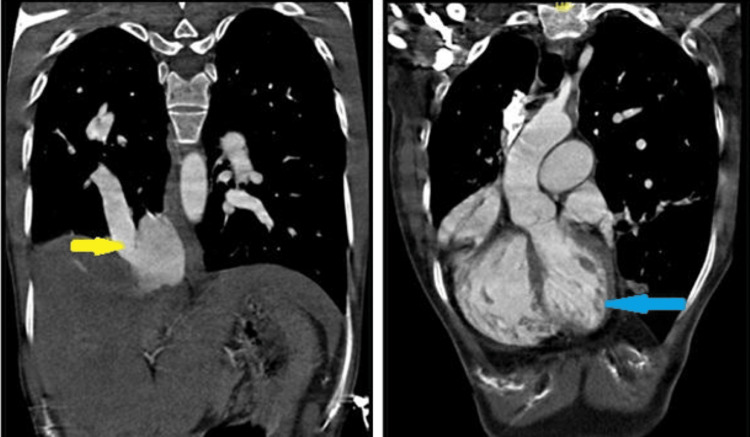
Coronal view of CT scan Left panel: Scimitar vein opening at IVC-RA junction (yellow arrow); Right panel: Dextro-posed heart shifted into right hemithorax (blue arrow) IVC: Inferior vena cava, RA: Right atrium

On admission, the general physical examination was unremarkable except that the apical impulse was palpable in the 4th intercostal space in the right midclavicular line and there was a mid-diastolic murmur. The chest X-ray showed a characteristic scimitar vein shadow, associated with a hypoplastic right lung and a dextro-rotated heart (Figure 1). Transthoracic echocardiography also revealed a single right pulmonary vein draining into the IVC-RA junction, moderate mitral stenosis, mild mitral regurgitation, and mild pulmonary artery hypertension (Figure 2). A CT scan demonstrated a dextro-rotated heart, a hypoplastic right lung, and an anomalous right pulmonary vein that drained into the IVC-RA junction (Figure 3).

After multidisciplinary discussion, scimitar vein rerouting with mitral valve repair or replacement was planned. A key dilemma was whether the moderate mitral stenosis requires a concomitant intervention, as rerouting could increase pulmonary venous return and worsen the mitral stenosis gradient. Surgery was performed through median sternotomy under cardiopulmonary bypass.

**Figure 4:**
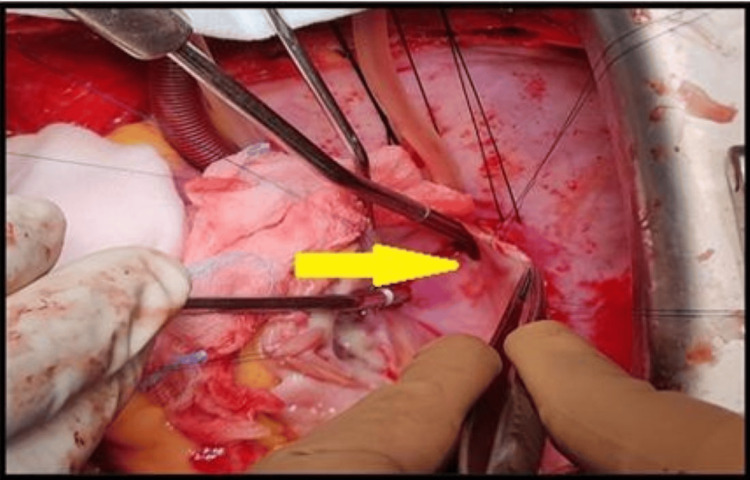
Intraoperative view of scimitar vein opening at the IVC-RA junction (yellow arrow) IVC: Inferior vena cava, RA: Right atrium

**Figure 5:**
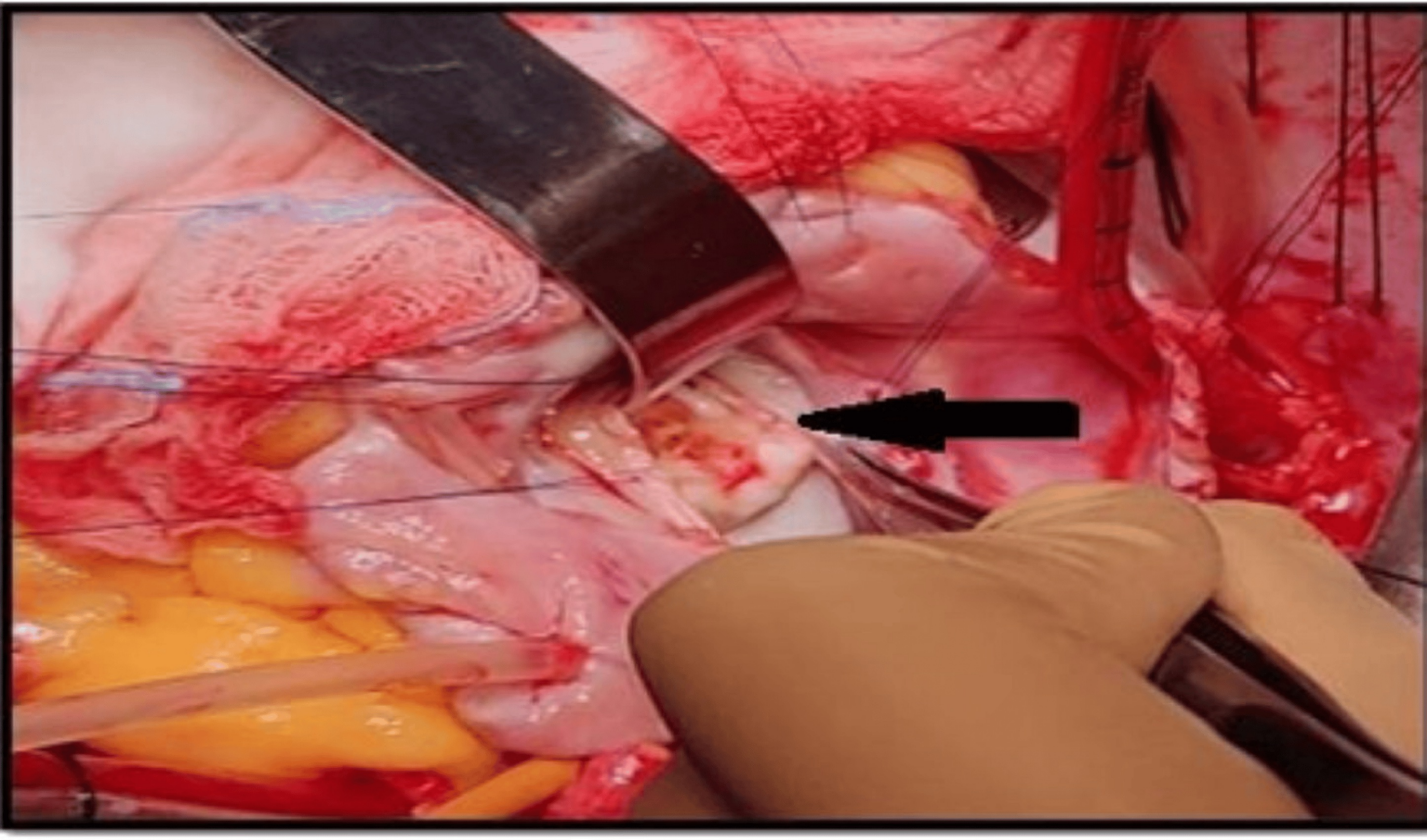
Intraoperative view of thickened and calcified mitral valve leaflet (black arrow)

Intraoperatively, the heart was dextro-rotated, with a single right pulmonary vein entering the IVC-RA junction (Figure 4). The mitral valve was rheumatic, with thickened anterior and posterior leaflets, everted leaflet edges, and calcification of the P1-P2 segments, producing both stenosis and regurgitation (Figure 5).

Preoperative evaluation revealed a moderate mitral valve gradient. However, intraoperatively, the valve appeared too damaged to repair, with thickening, retraction, and calcification of both leaflets. Hence, a 25 mm mechanical valve was initially placed, with the larger orifice facing the greater aortic curvature. However, during testing, the leaflet contacted the ventricular free wall due to the altered orientation, in contrast to the normal heart. The valve was then rotated clockwise by a few degrees to a more appropriate orientation. The valve opening was reconfirmed with a tester and saline inflation. The scimitar vein was then rerouted using pericardium.

Postoperative recovery was uneventful, and the patient was discharged on the fifth postoperative day. The patient followed up in the outpatient department. At the third-month follow-up, she was asymptomatic and in functional class I.

## Discussion

Scimitar syndrome arises embryologically from a failed connection between the right lung bud and pulmonary venous outpouchings, resulting in anomalous systemic venous return via the scimitar vein [3]. Clinically, SS presents either as a severe infantile form with a mortality rate up to 45% or as a milder childhood/adult form [7].

Radiological evaluation remains the mainstay of diagnosis. Cardiac catheterization assesses shunts, pulmonary artery hypertension, and anomalous systemic arteries for potential embolization. Surgical intervention is indicated in symptomatic adults, patients with recurrent infections, asymptomatic patients with a significant left-to-right shunt (Qp/Qs > 1.5), or when associated with a cardiac anomaly [5]. An infant with a large left-to-right shunt, severe pulmonary artery hypertension, heart failure, and particularly cyanosis requires surgical correction. However, surgical morbidity is notably high when the procedure is performed under the age of one year [8].

Our case was further complicated by the presence of RHD. The altered orientation of the atrium and mitral valve makes exposure difficult. Careful adjustment of the prosthetic valve orientation was required to prevent leaflet obstruction by the ventricular wall.

## Conclusions

Scimitar syndrome is often found incidentally, but its occurrence with RHD is even more rare. Management depends on the severity of valve disease and the presence of symptoms. Mitral valve replacement in SS poses significant surgical challenges due to abnormal anatomy. Thorough preoperative evaluation, multidisciplinary planning, and understanding of cardiac physiology are essential to avoid worsening mitral valve gradients.
